# Supplementary material for: Novel Competitive ELISA Utilizing Trimeric Spike Protein of SARS-CoV-2, Could Identify More Than RBD-RBM Specific Neutralizing Antibodies in Hybrid Sera
Source: Vaccines (Basel). 2024 Aug 13;12(8):914. doi: 10.3390/vaccines12080914 (PMC11359269; doi:10.3390/vaccines12080914)
Supplement: Supplementary file 1 [file vaccines-12-00914-s001.zip › vaccines-3086405-supplementary.pdf]

vaccines

MDPI

Supplementary Materials

Novel Competitive ELISA Utilizing Trimeric Spike Protein of SARS-CoV-2, Could Identify More than RBD-RBM Specific Neutralizing Antibodies in Hybrid Sera

S2. Supplementary Material and Methods

S2.1. Sera Donors

The study was approved by the Institutional Review Board of Hellenic Pasteur Institute (protocol code 7345 and date of approval 23 June 2021). In summary, the main inclusion criteria for the sera donors included: (i) signed informed consent; (ii) confirmation of vaccination with the appropriate dose (1st, 2nd, and 3rd dose) of Pfizer vaccine (BNT162b2, BioNTech/Pfizer); (iii) the time interval between vaccination and the subsequent blood donation being 21—25 days.

S2.2. Antigen S(6P) Trimer Conformation

We evaluated the eluted protein in an analytical run by gel filtration analysis using the Superose 6 Increase 10/300 GL column (GE Healthcare, Sweden) on an Akta FPLC purifier.

S(6P) trimer formation was detected in the final eluted product material of the anti-strep column purification. The eluted protein was analyzed by gel filtration analysis using the Superose 6 Increase 10/300 GL column (GE Healthcare, Sweden) on an Akta FPLC purifier. As shown in Figure S1A, the main peak of the graph corresponded to a molecular mass between 669 and 440 KDa. This is a strong indication that the protein was indeed expressed mainly as a trimer, since the theoretical molecular mass of the monomer is 144 KDa and the protein expressed is glycosylated.

S(6P) of the eluted protein of the anti-strep column purification performed on a 4–20% gradient precast gel (Nippon Genetics). An image of the gel showed a high purity and homogeneity of the used material (Figure S1B).

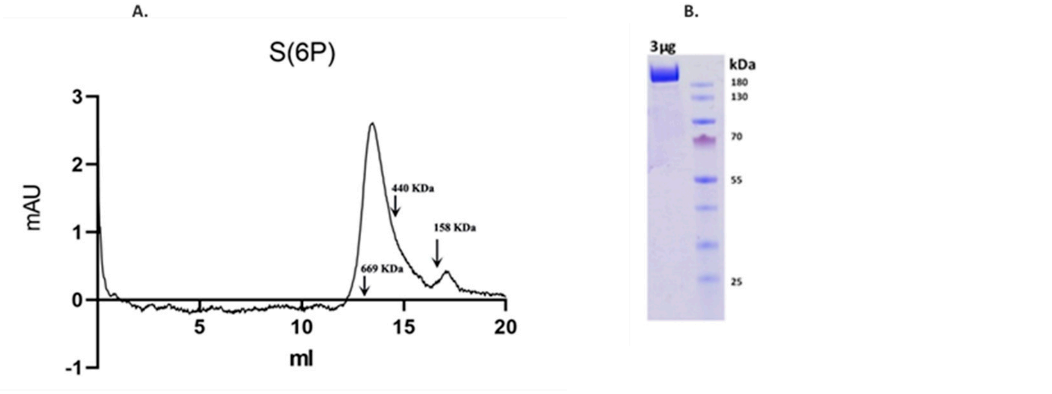

Figure S1. (**A**) Gel filtration analysis of the expressed protein S(6P). The markers indicate the elution volumes of protein markers with known molecular weight (thyroglobulin: 669 KDa, ferritin: 440 KDa, and aldolase: 158 KDa). Absorbance was detected at 280 nm and was expressed in mAU. The main peak of the eluted protein corresponds to the molecular mass of a trimeric state. (**B**) S(6P) purified protein performed on a 4–20% gradient precast gel.

S2.3. Antigens Quantification and Storage Conditions

Recombinant protein Antigens (Ags) were quantified using the A_{280} method as follows:

Absorbance at λ_{280}

Concentration = -- × Molecular weight × Dilution factor
Extinction coefficient* × Path-length

Extinction Coefficient and Molecular weight of recombinant (r) protein Ags

r-ACE2 = (156,290 M^{-1} cm^{-1} and 72.09 kDa) and r-trimeric S(6P) = (440,535 $M^{-1}cm^{-1}$ & 424.238 kDa)

Recombinant proteins were divided into aliquots and stored at −80 °C to avoid freeze–thaw cycles. Spike protein was also appropriately diluted in FBS at a final volume of 20μl and stored at −80 °C, as this approach was giving it strong antigenic stability. We tested the ability of these S(6P)-FBS samples to interact with immobilized ACE2 after 2 years of storage at −80 °C, compared to S(6P)-PBS samples stored for 2 years at −80 °C and S(6P) newly expressed and purified protein. Results from ELISA experiments showed exactly the same antigenicity characteristics between freshly purified samples and S(6P)-FBS samples, in contrast to S(6P)-FBS and S(6P)-PBS samples, while less antigenicity was achieved with S(6P)-PBS samples (data not shown). The ACE2 protein was the most sensitive molecule, and after six to eight months its binding sensitivity to the S(6P) trimer decreased. Six expression experiments for ACE2 were performed to complete the experiments and the products of two of them were rejected as inappropriate.

S2.4. Inhibition ELISA Strategy

Followed development steps:

(a) Recombinant hACE2 protein diluted in PBS (pH 7.2) was coated (2.75 μg/ml, 50 μL/well except where otherwise stated) in 96–well half-area high binding plates (Corning 3690) O.N. at 4 °C. Following this, the plates were treated with three washing steps (0.1% (*v*/*v*) PBS-Tween-20; pH 7.2), and non-specific binding sites were blocked with either 50% (*v*/*v*) FBS(-IgG) in PBS-T (0.1%), (FBS solution), or 5% n.f.-milk in PBS (filtered), (milk solution) for 1.5h at room temperature (R.T). After blocking, the same washing steps described above followed. Different concentrations of S(6P), mAb anti StrepTag (StrepMAB-Immo, iba) at a final dilution of 1:2500 and anti-mouse/HRP (final dilution 1:10,000), (#31439, ThermoScientific), diluted in the FBS solution or n.f.-milk solution (3% n.f.-milk in PBS-T(0.1%), filtered), were mixed in low binding Eppendorf tubes and pre-incubated for 1h at 37 °C. Consequently, 70 μL of each mixed sample was transferred to a low-binding plate and then 50 μL was added simultaneously in technical duplicate or triplicate to the above ACE2 coated ELISA plate for 1h at R.T. Unbound material was removed by four washing steps (three washes with PBS-T(0.1%) and one wash with PBS). The chromogenic reaction was quantified following the addition of 50μl TMB substrate reagent (TMB, #34021, ThermoScientific) and 50μl stop solution (1M H_2SO_4). The absorbance of the samples was measured at 450nm on a microplate reader (Bio-Rad, Hercules, CA, USA). All measurements were carried out in duplicate or triplicate and averaged.

(b) Antigen trimeric S(6P) at a concentration of 195ng/ml PBS, was used (50 μL per well) to coat half-area 96–well high binding plates, O.N. at 4 °C. The plates were washed twice with washing buffer, and non-specific binding sites were blocked with the appropriate blocking solution for 1.5h at R.T. The blocking solution was discarded and the plates were washed twice with washing buffer. Serum samples from vaccinated people were two-fold serial diluted in low binding Eppendorf tubes in the appropriate solution, transferred to a low-binding plate, and then 50 μl was added simultaneously to the above ELISA plate in duplicate or triplicate for 1h at 37 °C. Unbound serum proteins were removed by four washing steps (three washes with PBS-T (0.1%) and one wash with PBS) and goat anti-human Fcγ Abs -conjugated HorseRadishe Peroxidase (HRP) antibody (1:20,000 in the appropriate solution), (3143, ThermoScientific), was added for 1h at 37 °C. For the detection of peroxidase activity, the plates were washed three times with washing buffer and once with PBS, and TMB substrate reagent was added for 15–20 min. The reaction

was stopped with 1M H_2SO_4, and the absorbance was measured at 450 nm on a microplate reader). All measurements were carried out in duplicate or triplicate and averaged.

(c) Recombinant hACE2 protein diluted in PBS (pH 7.2) was coated (2.75 μg/ml, 50 μL/well except otherwise is referred) in 96–well half-area high-binding plates (#3690, Corning) O.N. at 4 °C. The plates were washed twice with washing buffer, and non-specific binding sites were blocked with FBS solution 1.5h at R.T. The same solution was used as diluent for the following steps of the ELISA assay. In some experiments a blocking solution of 5% n.f.-milk in PBS and a diluent of 3% n.f.-milk in PBS-T (0.1%) were used.

A final concentration of trimeric-S(6P) 115 ng/ml (unless otherwise is referred) and final dilutions of 1:10,000 of anti-mouse/ Fcγ/HRP and 1:2500 anti-Strep-tag were mixed together in low binding Eppendorf tubes, gently vortexed and pre-incubated with serum sample for 1h at 37 °C (final volume of 240 μl). Mixed samples (70 μL) were transferred to a low-binding plate and then 50 μL was added simultaneously in technical duplicate or triplicate to the above ACE2 coated ELISA plate for 1h at R.T. Unbound material (complexes of S(6P)-strep/tag-conjugate/HRP) was removed by four washing steps (three washes with PBS-T(0.1%) and one wash with PBS). For the detection of peroxidase activity, 50 μL of TMB substrate reagent was added for 15–20 min. The reaction was terminated with 50 μL of 1M H_2SO_4, and the absorbance was measured at 450 nm on a microplate reader (Bio-Rad, Hercules, CA, USA). All measurements were carried out in duplicate or triplicate and averaged.

For the calculation of the percentage of inhibition, we used the equation: Inhibition (%) = (1 − (sample optical density value / negative control optical density value)) × 100. Negative control is defined as the sample without sera, applied in at least four to six wells in different places of the plate, whereas two positive controls (high: 92–96% and medium: 40–50% of inhibition in 1:20 serum dilution) where always used. Importantly, the absorbance of the blank samples (without S protein) and background samples (without coated ACE2) were always less than 0.080 at 450 nm, independently of the developing time with TMB substrate.

For positive and negative serum validation, a final 1:20 dilution of the test serum was used. For determination of neutralization titers, serum samples were used with a twofold serial dilution starting at 1:20. The same dilution range from 1:20 to 1:2560 was used to facilitate side-by-side comparison in the correlation studies of the two different tests (sVNA and cPass of GenScript).

S2.5. Surrogate Virus Neutralization Test Kit (c-Pass Assay, GenScript)

Sera samples were tested for neutralizing activity using the SARS-CoV-2 Surrogate Virus Neutralization Test Kit (sVNT), (cPass Assay, GenScript) as per the manufacturer's instructions. We used the latest cPass version with the cutoff defined at 30% inhibition. Samples and provided positive and negative controls were diluted 1:10 with provided Sample Dilution Buffer, while consecutive dilutions were applied for the titration of the neutralizing activity. A total of 125 μL of sample/control was mixed 1:1 with RBD-HRP solution and incubated at 37 °C for 30 min. Mixed samples/control (120 μL) were transferred to a low-binding plate and then 100 μL of each sample/control was simultaneously added to the provided hACE2 coated plate in technical duplicate and incubated at 37 °C for 15 min. Wells were then washed 4 times with 200 μL of provided Wash Solution. 100 μL provided TMB solution was added per well and the plate was incubated at room temperature for 15 min. A total of 50 μL of the provided Stop Solution was added per well to terminate the reaction, and absorbance at 450 nm was read immediately on a microplate reader. Data was analyzed as per the manufacturer's instructions. Relative inhibition was calculated by the equation Inhibition (%) = (1 – (OD value of Sample Mean/OD value of Negative Control)) × 100. According to the

manufacturer's instructions, values ≥30 are considered positive for neutralization, whilst those < 30 are considered negative.

S3. Supplementary Results

S3.1. Optimizing the surrogate Virus Neutralization Assay (sVNA)

We studied the effect of n.f.-milk (non-fat milk) and FBS on the interaction of S(6P)-StrepTag molecules with ACE2, as well as between specific h-IgG antibodies from sera of vaccinated individuals and this antigen. The optimal concentration of immobilized ACE2 was determined to be 2.75 μg/ml in a 50 μL volume/well (138 ng/well) (Figure S2). Figure S3A shows that binding of S(6P)-StrepTag antigen at the immobilized hACE2 was specific and dose dependent when both agents were used. Notably, the observed binding was higher when FBS was used instead of n.f.-milk (Figure S3A).

In contrast, the binding of specific IgG Abs from sera of vaccinated individuals to immobilized S(6P) was the same with both agents (data not shown). We did not investigate further this negative effect of n.f.-milk on ACE2:S(6P)-StrepTag interaction and chose FBS to complete the experiments.

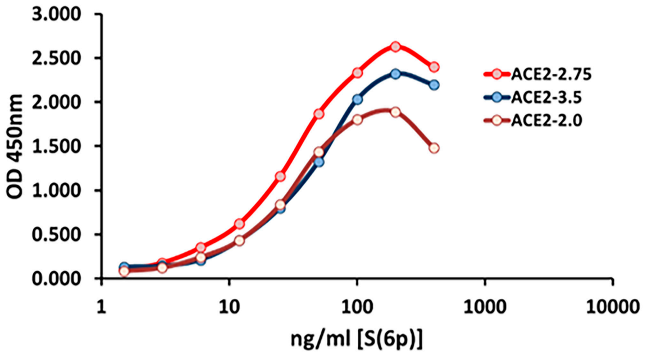

Figure S2. Direct binding of SARS-CoV-2 trimeric Spike S(6P)-HexaPro to different concentrations of hACE2 immobilized on the ELISA plate, using FBS reagent. Concentration of 2.75 μg/ml (50 μL/well, 138 ng/well) was chosen over the other two. Representative of three independent experiments with each concentration response tested in duplicate. Dots represents mean values from these experiments.

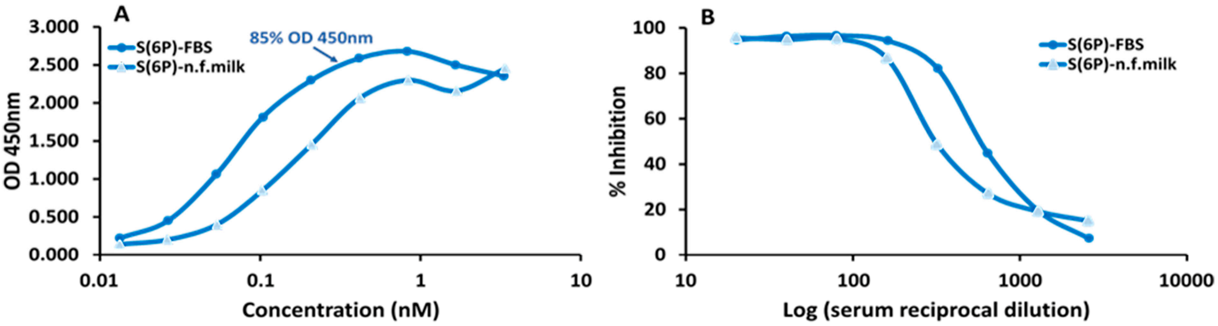

Figure S3. (**A**) Binding of S(6P)-StrepTag to hACE2 immobilized on ELISA plate using FBS (blue) and n.f.-milk (open symbols) agents. Representative of three independent experiments, with each concentration response tested in triplicate. Dots represent mean values from these three replicates. (**B**) Comparison of the sVNA inhibition titration curves when S(6P)-StrepTag combined with FBS and n.f.-milk interacts with nAbs of serum dilutions. Representative of three independent experiments with each dilution response tested in duplicate. For this experiment a serum S7 was used.

Subsequently, we proceeded a step further to use S(6P)-StrepTag in combination with the two agents to compare the final inhibitory effect, when testing serum from vaccinated

individuals. The concentration of S(6P) corresponding to approximately 80–85% of the maximum absorbance of the relative binding curve was determined as appropriate (optimal concentration) for interaction with the nAbs in the pre-incubation step of the assay. Three independent experiments resulted in an optimal mean concentration of 115 ng/ml (0.27 nM) and 178 ng/ml (0.42 nM) of trimeric S(6P)-StrepTag in FBS and n.f.-milk solutions respectively (Figure S3B). The S(6P) antigen at optimal concentrations, and both agents (FBS and n.f.-milk solutions), were combined for the development of the respective inhibition curves produced from vaccinated individuals' sera.

As shown in Figure S3B, in a final comparative exposition, the utilization of S(6P) in combination with FBS performed better producing a more desirable inhibition curve than n.f.-milk. This result was confirmed in a larger number of sera of vaccinated subjects. In addition, the use of n.f.-milk instead of FBS combined with S(6P) usually resulted in divergence from the norm of the lower part of the titration curves (Figure S4A–D). Furthermore, a lower intra-assay reproducibility of inhibition values was observed using S(6P)-StrepTag compared to n.f.-milk (data not shown).

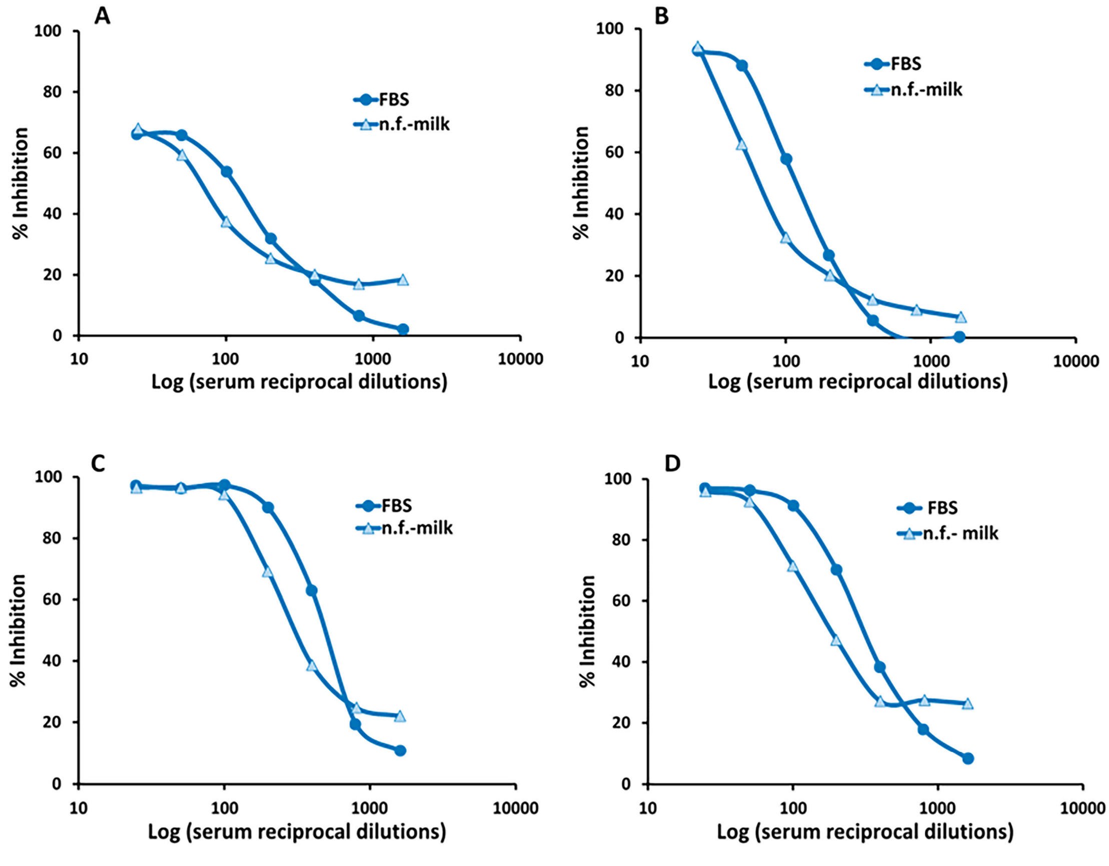

Figure S4. Inhibitory titration curves of the sVNA assay, when S(6P)-StrepTag combined with FBS and n.f.-milk solutions. For this experiment, two S7 and two S8 sera were used (serial dilutions from 1:25 to 1:1600). Dots represent mean values of duplicate dilution responses.

S3.2. Testing Sera Samples with sVNA and cPass

International standard (NIBSC (21/234)

We tested the inhibitory effect against the WHO international standard for anti-SARS-CoV-2 immunoglobulin (NIBSC (21/234) twice with both assays, in independent experiments. As a result, we generated two "median" comparable inhibitory curves (the % inhibition for each serum dilution was estimated and averaged for the creation of the "median" inhibitory curve) from the sVNA and cPass assays, with mean ID_{50} values of 267.5 and 293.8, respectively.

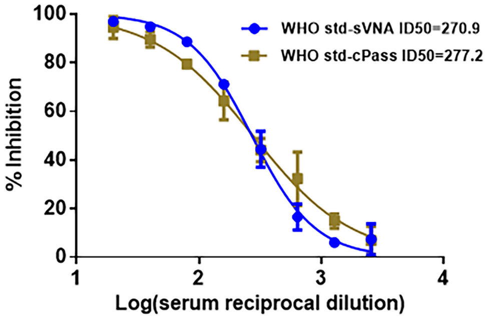

Figure S5. Titration curves of sVNA and cPass. Percent inhibition of serial dilutions of the WHO international standard for anti-SARS-CoV-2 immunoglobulin (NIBSC (21/234)*, tested in two different experiments, in duplicate, with sVNA and cPass assays. Each point represents the average of the mean ± SEM of the two experiments. *Pooled plasma samples obtained from individuals recovered from COVID–19, collected between April–May 2020.

Sera from a Vaccinated Individual

Inhibitory curves of all sera samples throughout the vaccination period of a person that was infected with SARS-CoV-2 seven months after third dose of the vaccine. All ID_{50} values of samples obtained after 2nd and 3rd dose administration of the vaccine were gradually weakened. Interestingly, the inhibitory dilution titer values that go with (ID_{50}) are obviously higher after the 3rd vaccine dose (brilliant blue line) and even more so after COVID-19 (S11: 25 days after PCR confirmation for SARS-CoV-2).

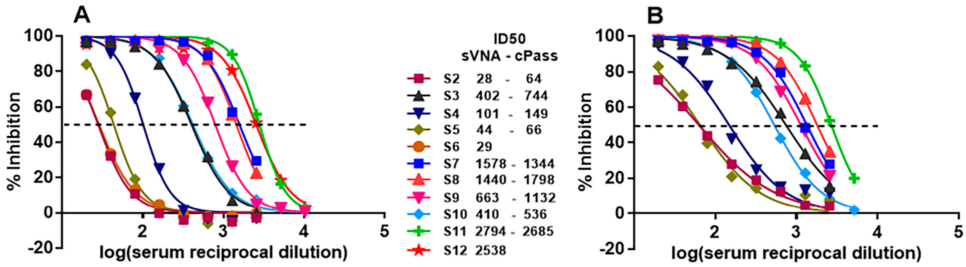

Figure S6. Titration curves of sVNA and cPass. Percent inhibition of serial dilutions of sera samples S2–S12 (please see Material and Methods, Table 1) of a vaccinated individual that was infected with SARS-CoV-2, tested by (**A**) sVNA and (**B**) cPass assays. ID_{50} values were determined as described in the method section, and are presented here. The dotted lines indicate the ID_{50} inhibition level.

Sera from fully vaccinated individuals who contracted COVID-19 (hybrid sera)

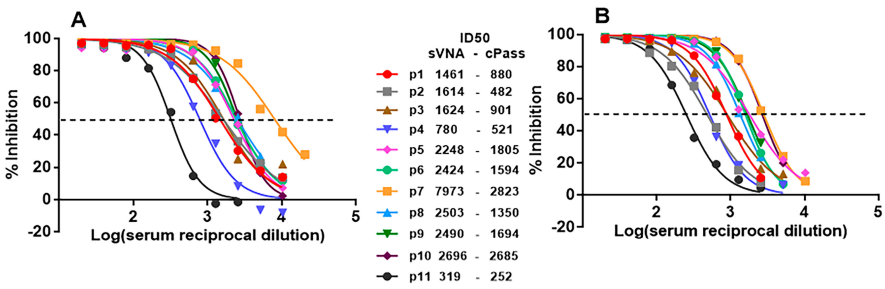

Figure S7. Titration curves of sVNA and cPass. Percent inhibition of serial dilutions of sera samples from eleven vaccinated patients with COVID-19 (hybrid sera) tested with (**A**) sVNA and (**B**) cPass, and the determination of ID_{50} values. The dotted lines indicate the ID_{50} inhibition level. Eight of these samples were performed with sVNA on the same ELISA plate simultaneously with sera from vaccinated subjects (see Results 3.4, Table 2).
